# A Moderated Mediation Model Explaining the Relationship Between Risk-Group Membership, Threat Perception, Knowledge, and Adherence to COVID-19 Behavioral Measures

**DOI:** 10.3389/fpubh.2022.842368

**Published:** 2022-05-19

**Authors:** Sebastian Sattler, Shannon Taflinger, André Ernst, Fabian Hasselhorn

**Affiliations:** ^1^Faculty of Sociology, Bielefeld University, Bielefeld, Germany; ^2^Institute of Sociology and Social Psychology, University of Cologne, Cologne, Germany; ^3^Pragmatic Health Ethics Research Unit, Institut de Recherches Cliniques de Montréal, Montréal, QC, Canada; ^4^GESIS – Leibniz Institute for the Social Sciences, Cologne, Germany

**Keywords:** public health, COVID-19, risk group membership, perceived COVID-19 threat, adherence to COVID-19 measures, knowledge, non-pharmaceutical intervention

## Abstract

**Background:**

COVID-19 is a threat to individual and global health, thus, reducing the disease's spread is of significant importance. However, adherence to behavioral measures against the spread of COVID-19 is not universal, even within vulnerable populations who are at higher risk of exposure to the virus or severe COVID-19 infection. Therefore, this study investigates how risk-group membership relates to adherence to COVID-19 behavioral measures, whether perceived threat of COVID-19 is a mechanism explaining this relationship, and whether knowledge about COVID-19 moderates these effects.

**Methods:**

We conducted a web-based survey (*N* = 4,096) representative of the adult population in Germany with regard to gender, age (18 to 74), and province. Therein, we assessed risk group membership with two indicators (risk of exposure to COVID-19 and risk of severe COVID-19 infection), perceived COVID-19 threat with the Perceived Coronavirus Threat Questionnaire, knowledge about COVID-19 with a knowledge test; and adherence to six behavioral measures to protect against the spread of COVID-19 (e.g., keeping distance, using mouth-nose protection, and following contact restrictions). We used moderated mediation models to test whether perceived threat mediates the relationship between risk-group membership and adherence and whether knowledge about COVID-19 moderates this relationship.

**Results:**

We found that risk group members had more perceived COVID-19 threat and that knowledge about COVID-19 increased perceived threat. Moreover, risk group membership had a positive direct effect on adherence to most behavioral measures and risk group members with less knowledge about COVID-19 violated measures more frequently. Risk-group membership also had positive indirect effects on adherence via perceived COVID-19 threat. The moderated indirect effects of threat indicate that threat led to more adherence when knowledge was low, but lost relevance as knowledge increased.

**Conclusion:**

The results may help to evaluate disease-regulation measures and to combat the pandemic more effectively. For example, increasing COVID-19 knowledge in the general population could increase adherence to COVID-19 behavioral measures. However, policy makers should be mindful that this could also have negative mental health implications as knowledge increases perceived COVID-19 threat.

## Introduction

Since its emergence in 2019, the coronavirus disease (COVID-19) has threatened the lives of individuals around the globe. As of July 1, 2021, there have been over 274 million confirmed cases of COVID-19 and 5.3 million deaths worldwide (1). Furthermore, approximately 20% of those infected experience persistent health problems, such as damaged lungs, depression, and fatigue (2). While there is still much unknown about the long-term health consequences of COVID-19, the World Health Organization has recognized “Long COVID”, a long-lasting and debilitating condition marked by symptoms such as shortness of breath and cognitive dysfunction (3).

Until recently, there were no vaccines to prevent COVID-19. Therefore, many countries have mandated wearing masks in public spaces; closed stores, restaurants, schools and national borders; and in severe cases, only allowed citizens to leave their homes for essential purposes (4). The social and economic costs of such measures include, but are not limited to, negative impacts on student learning (5) and a sharp decrease in working hours and job losses (6). Consequently, countries have spent billions of dollars to support their economies and healthcare systems (7, 8).

Lingering vaccination hesitancy (9), upcoming virus variants (10), and the failure of essential antibodies to form in 20 percent of COVID-19 infections (11) necessitate behavioral measures to slow the spread of the virus and prevent further deaths and suffering. Such measures include mask mandates, hygiene recommendations, and contact restrictions. Although behavioral measures have lower costs than widespread lockdowns, adherence is not universal within and across countries (12). Failure to comply with preventative behavioral measures has been associated with increased COVID-19 infections (13). Therefore, it is imperative that governments understand the motivations of adherence to behavioral measures in order to be able to develop efficient health communication strategies for the current and potential future pandemics (14).

The adherence of individuals at risk of exposure to the COVID-19 virus (e.g., essential workers in a hospital or school) or at risk of severe COVID-19 infection (e.g., suffering from diabetes or a lung or heart disease) is of particular interest. The first group may be more likely to become infected and infect others (due to their socio-structural position), whereas the second group is more likely to suffer from detrimental, and potentially lethal, health consequences in case of an infection. Studies concerning the relationship between being at increased risk and adherence to preventative behavioral measures reveal mixed results: positive (12, 43), nonsignificant (15) as well as negative effects (15, 16). One likely reason for the negative effects of risk of exposure (essential worker status) on adherence could be that these studies focused on one behavioral measure, physical distancing, which is not feasible for many essential workers. To deepen our understanding of the relationship between risk group membership and adherence as well as the conditions under which such a relationship might be modulated, research needs to examine a broader range of behavioral measures and use a sample of the general population.

Given the possibility for future harms and losses that individuals at risk of exposure or severe infection face (e.g., spreading the virus to others and hospitalization or death upon contracting the virus), it is likely that individuals who recognize their high-risk status appraise COVID-19 as a threat. Such a threat appraisal often, in turn, induces feelings of anxiety, fear, and worry (17). In accordance, frontline healthcare workers, who work directly with COVID-19 patients and are at increased risk of exposure to the virus, have higher anxiety levels than non-frontline healthcare workers (18, 19). Also, individuals with poorer self-rated health, hence at increased risk of severe COVID-19 infection, have more stress and anxiety (20). More generally, several studies have found a relationship between perceived risk and anxiety or fear related to COVID-19. For example, Lin et al. (21) discovered that individuals who have higher perceived severity and susceptibility of COVID-19 have more anxiety, while Sloan et al. (22) established an association between perceived risk of dying and personal fear of COVID-19, and Winter et al. (23) demonstrated a positive association between perceived vulnerability to disease and fear of COVID-19.

In this process, anxiety can be assumed to be an adaptive response that helps individuals detect and protect themselves from potential threats (24). In the context of the COVID-19 pandemic, COVID-19-related anxiety may similarly prompt avoidance of situations where infection is likely or induce the uptake of preventative measures. This can be seen as a form of problem-focused coping (17) as it reduces the likelihood of contracting and spreading the virus. Several studies have also found relationships between anxiety, worry, fear, and behavior change or adherence to COVID-19 behavioral measures (14, 23, 25). Therefore, it stands to reason that individuals who perceive themselves at risk feel threatened by the virus, prompting them to adhere to behavioral measures, as supported by first evidence (26). Given the prior findings on the relation between being at risk and adherence, being at risk and anxiety, as well as anxiety and adherence in the context of COVID-19, we explore: first, whether individuals at risk of exposure to COVID-19 or severe COVID-19 infection adhere more often to COVID-19 behavioral measures and, second, whether perceived threat of COVID-19 mediates the relationship between risk group membership and adherence.

To better understand how risk group membership affects adherence via perceived COVID-19 threat, we examine the moderating role of knowledge about COVID-19. Thereby, one question is whether perceived threat is necessary to induce adherence to the recommended COVID-19 behavioral measures if knowledge is high. This potential moderation is of interest because knowledge is a modifiable characteristic and can potentially be altered with interventions, such as public health campaigns (27).

In the context of the COVID-19 pandemic, knowledge about the virus is associated with increased adherence to behavioral measures (28, 29) and increased perceived efficacy of protective behaviors (30). As increased perceived efficacy of coping options lowers threat perception (17), individuals who understand the virus, its symptoms, consequences, and the ways which they can protect themselves likely have less feelings of anxiety or worry related to COVID-19 that arise as a result of their risk group membership. Tan et al. (31) has similarly suggested that knowledge about the virus could play a role in fear reduction in explaining the increased anxiety in non-medical healthcare workers compared to medical healthcare workers. Thus, we will examine whether knowledge about COVID-19 reduces the effect of risk group membership on perceived COVID-19 threat.

Knowledge about COVID-19 may also moderate the effect of perceived COVID-19 threat on adherence. Such effects are also known from other contexts; for example, Nabi et al. (32) found that the effect of fear on intended self-examination for early cancer detection was higher for individuals with low subjective knowledge about cancer. However, the effect of fear on behavior was greatly reduced at higher levels of subjective knowledge. Thus, one question is whether anxiety is not always needed to induce compliance, and whether there are conditions, such as increased knowledge, which similarly spur behavioral change. Consequently, we will explore whether knowledge about COVID-19 moderates the relationship between perceived COVID-19 threat and adherence to behavioral measures.

In addition, we examine whether any remaining direct effects on adherence (not mediated by perceived COVID-19 threat) vary with knowledge about COVID-19. As previous research has shown that having read public health information on recommended behaviors to protect oneself is associated with increased behavioral change, but not increased anxiety (33), risk group members who have more knowledge about COVID-19 may adhere to measures more often, without having increased perceived COVID-19 threat. Furthermore, knowledge about COVID-19 is related to perceived outcome efficacy of the behavioral measures (30), thus risk group members with more knowledge about COVID-19 may believe that the behavioral recommendations are more effective and therefore choose to engage in these behaviors without having increased perceived COVID-19 threat. Consequently, we want to also explore whether the direct effect of risk group membership on adherence is moderated by knowledge about COVID-19.

### Aims of This Study

**Figure 1:**
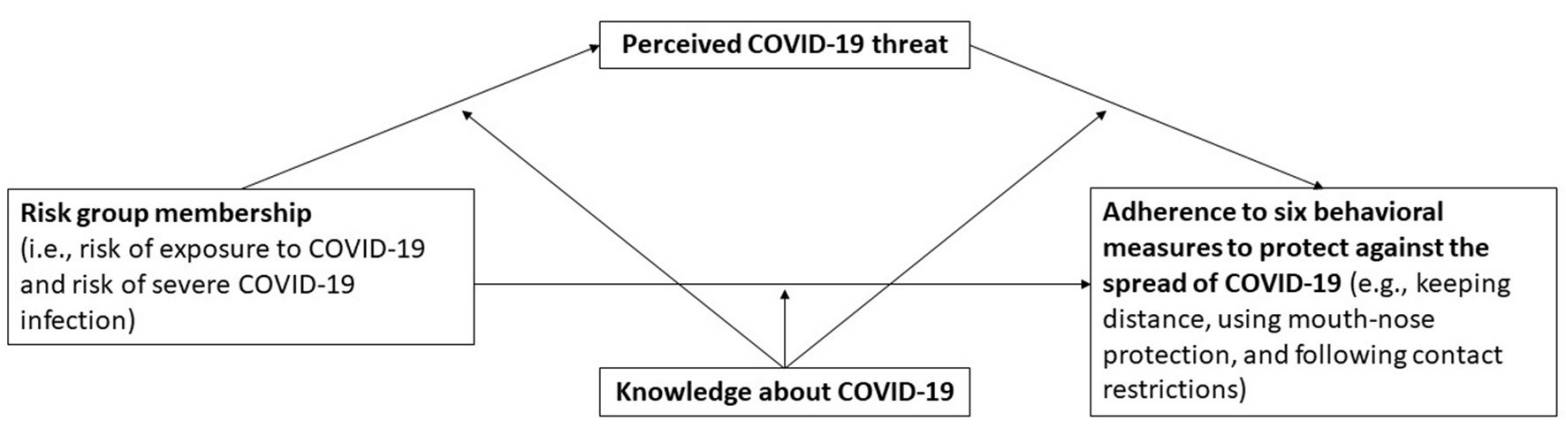
Overview of the explored relations.

Our study aims to understand how risk group membership affects adherence to an array of six COVID-19 preventative behaviors recommended by the Robert Koch Institute (RKI), which is a federal institution central to the field of disease monitoring and prevention in Germany. We focus on two classifications of risk, being at risk of exposure and at risk of severe infection. To this aim, we conducted a web-based study with a large sample representative of the adult population of Germany. We developed and tested a moderated mediation model which explores the relationship between risk group membership and adherence to COVID-19 behavioral measures and whether this relationship is mediated by perceived COVID-19 threat and further moderated by knowledge about COVID-19 (see Figure 1 for an overview of the explored relations).

## Methods

### Participants and Design

We recruited 4,856 adult participants living in Germany to participate in an online survey via the survey provider *respondi Online Panel*, an actively managed panel used for market research with voluntary participation and a double opt-in registration process.^1^ Respondi panel members were invited via an email in which the topic was not mentioned, which can reduce selective survey uptake due to topic salience. Data were collected between December 16–29, 2020; when Germany was under a lockdown that closed non-essential stores and services. To increase representativity, we used a quota sample representative of the German population with regard to sex, age (18–74), and province. Following German data protection regulations, personal data and survey data are stored separately. Of the 4,856 respondents, 4,716 participants (97.1%) provided informed consent and, therefore, were eligible for participation in the study. Due to missing data on the model variables, 4,096 participants comprise the analytical sample. Thereof, almost every second participant was female (49.4%), while the average age was 45.48 (median: 46). Respondents who completed the survey received a small incentive (€0.40). This study was reviewed and approved by Faculty of Management, Economics and Social Sciences at the University of Cologne (ethics approval number: 200015DM_extension).

### Measures

*Adherence to behavioral measures:* We assessed the extent to which individuals adhere to behavioral measures against the spread of COVID-19, in the form of contact restrictions enacted by the German government and the AHA+L+A rules recommended by the RKI (34), namely 1) *maintain distance* (where prescribed), 2) *use mouth-nose protection* (where prescribed), 3) *follow the hygiene rules* (e.g., disinfect hands), 4) *adhere to contact restrictions* (e.g., not meet more people than allowed), 5) *use a corona app*^2^, and 6) *air rooms regularly*. Responses were assessed on a seven-point scale from “never” [0] to “always” [7]. Similar preventative measures have been previously assessed [e.g., (30)].

*Risk group:* We used two indicators to measure self-assessed risk group membership and provided participants with examples to clarify the meanings of both indicators. The first indicator measured was higher risk of exposure to the virus, “Are you a member of a risk group due to your higher risk of exposure to the virus (e.g., because of working in a hospital or school)?” (15, 16). The second indicator measured was higher risk of a severe infection, “Are you a member of a risk group due to your higher risk of severe COVID-19 infection (e.g., because of suffering from diabetes or a lung or heart disease)?” (12, 15). Response options were “no” [0] and “yes” [1] in each case.

*Perceived COVID-19 threat:* We used five items of the Perceived Coronavirus Threat Questionnaire (35) to measure how threatened or worried individuals were about COVID-19 (sample item: “I am afraid of the coronavirus (COVID-19)”). Answers ranged from “does not apply at all” [0] to “completely applies” [7]. Reliability was good (Cronbach's α = 0.86). The items were averaged using STATA's “*rowmean*” function. To ease the interpretation and to allow for a better comparability of the effects, this variable has been standardized (i.e., by subtracting the mean from each value and dividing it by the standard deviation), resulting in a mean value of zero and a standard deviation of one.

*COVID-19 knowledge:* Respondents were asked eight yes-no questions pertaining to their knowledge about COVID-19, such as “Is dry cough a symptom of corona?” and “Is corona caused by a bacterium?” (see Supplementary Table 1 for all items). Correct answers were summed. Thus, the measure ranges from 0 to 8. This variable has also been standardized (see above).

### Statistical Analysis

We computed a series of moderated mediation models using the SPSS macro, PROCESS (Model 59) (36), to evaluate whether knowledge about COVID-19 negatively moderates the impact of risk group membership on perceived COVID threat and adherence, and moreover, the impact of perceived COVID threat on adherence (see Figure 1). Each definition of risk (risk of exposure or severe infection) and behavioral measure were examined separately, leading to a total of two mediator models (effect of risk group on perceived COVID threat) and twelve dependent variable models (effect of risk group on adherence via perceived COVID threat). When testing the indirect effects, 95% percentile bootstrap confidence intervals (*95% CI*_*Boot*_) (*N* = 10,000)^3^ and 95% *CI*_*Boot*_ were used, whereby a CI that does not include zero indicates a statistically significant effect. The conditional direct and indirect effects of risk group membership are calculated for three different values (“low” at 1 SD below the mean, “average” at the mean, and “high” at 1 SD above the mean for COVID threat and at the maximum observed value for knowledge about COVID-19^4^). We plotted the conditional effects and confidence intervals according to the Johnson-Neyman significance region technique (37, 38). Johnson-Neyman plots visualize interaction effects and depict the conditional effects of the main variable of interest (X) on the dependent variable (Y) across the full range of values of the moderator (Z). The plot shows the 95%-confidence interval above and below the predicted conditional effect, thereby indicating for which values of Z the effect of X is statistically significant. Effects are statistically significant if confidence intervals are positive or negative, thus excluding zero. Thereby, we explored the full range of knowledge about COVID-19.

We will first show findings for risk of exposure to COVID-19 as the explanatory variable, then for risk of severe COVID-19 infection. In each case, we begin with the mediator model, which analyzes the relation between the respective risk group and COVID-19 threat as well as how this relationship is moderated by knowledge about COVID-19. Then, we present dependent variable models examining the effects of each risk group and COVID-19 threat on adherence to behavioral measures and how this is moderated by knowledge about COVID-19. Thereby, we also investigate the conditional indirect effects. All models control for age and gender as previous research has shown that both can play a role in membership to each of the risk groups (39, 40), perceived threat and fear of COVID-19 (41, 42), as well as adherence to COVID-related behavioral measures (12, 43).

## Results

### Descriptive Findings

Respondents complied with mask requirements most often, followed by distance requirements (Table 1). Meanwhile, use of an app was the least frequently observed behavior. Almost one in five respondents (18.46%) perceived themselves as being at risk of exposure, while almost one in three (30.01%) perceived themselves as being at risk of a severe infection. While one in twenty (4.98%) were in the lowest percentile of COVID-19 threat, about twice as many where in the highest percentile (9.67%). More than every second respondent (51.83%) answered all knowledge questions correctly. Pairwise correlation coefficients demonstrate positive relationships between all of the behavioral measures examined (see Table 2). The interrelationships between the adherence measures were moderate, with the exception of the use of a corona app, which was only weakly related to the other measures.

**Table 1:** Descriptive information (*N* = 4,096).

	**Mean**	**Standard deviation**	**Min**	**Max**
Distance	6.26	1.172	0	7
Mouth-nose protection	6.65	0.994	0	7
Hygiene rules	5.97	1.496	0	7
Contact restrictions	6.07	1.484	0	7
Corona app	3.07	3.237	0	7
Airing rooms	5.68	1.674	0	7
Risk of exposure	0.18	0.388	0	1
Risk of severe infection	0.30	0.457	0	1
Knowledge about COVID-19 (standardized)	0.90	0.149	0	1
COVID-19 threat (standardized)	0.57	0.251	0	1

**Table 2:** Pairwise correlations (*N* = 4,096).

	**Pairwise correlations**				
	**1)**	**2)**	**3)**	**4)**	**5)**
1) Distance					
2) Mouth-nose protection	0.662**				
3) Hygiene rules	0.580**	0.479**			
4) Contact restrictions	0.635**	0.533**	0.562**		
5) Corona app	0.113**	0.086**	0.148**	0.153**	
6) Airing rooms	0.472**	0.386**	0.544**	0.504**	0.206**

*Pearson's coefficients are reported*.

** *p < 0.01*.

### Risk of Exposure to COVID-19

**Figure 2:**
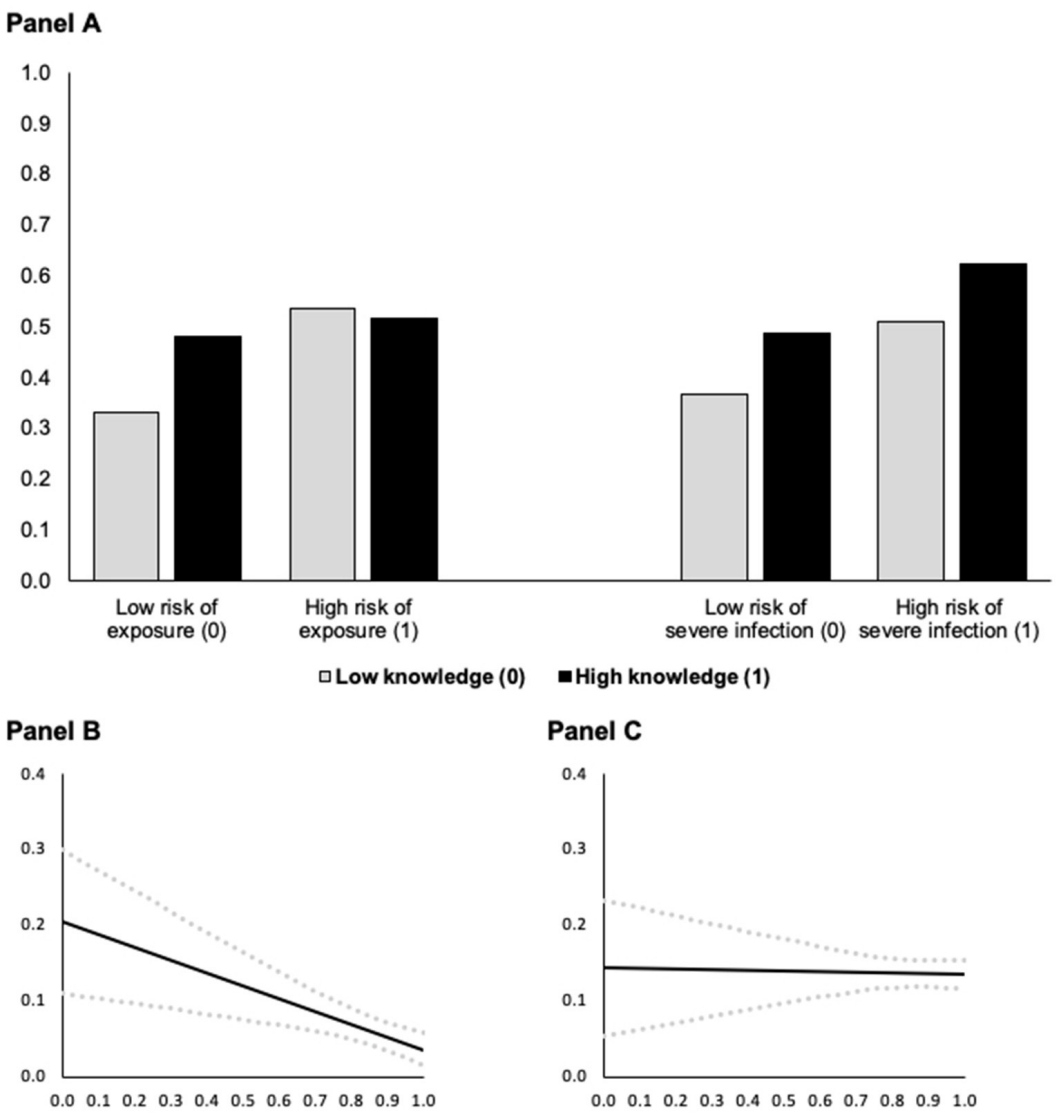
Predicted COVID-19 threat as a function of risk group membership **(A)** at different levels of knowledge about COVID-19 and conditional effect of risk of exposure **(B)** and of severe infection **(C)** on COVID-19 threat as a function of knowledge about COVID-19 (based on Models 1 and 2 in Table 3, *N* = 4, 096). In **(A)**, predictions are for men who are 18 years old. In **(B,C)**, the black solid lines (**—**) indicate the conditional effect of risk group membership from the lowest (0) to the highest (1) level of knowledge about COVID-19 and dotted gray lines (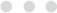) indicate the upper and lower 95% confidence interval; all Johnson-Neyman values are statistically significant (*p* < 0.05).

Model 1 in Table 3 shows the first mediator variable model, which describes COVID-19 threat as a function of risk group membership (risk of exposure) and knowledge and includes an interaction effect between risk group membership and knowledge. We found statistically significant positive conditional main effects of risk group membership and knowledge as well as a statistically significant negative interaction effect. Figures 2A,B illustrate the conditional effect of risk group at the minimum and maximum observed values of knowledge about COVID-19. They show that risk group membership increases COVID-19 threat but this effect decreases with knowledge about COVID-19. Panel B indicates that the effect of risk group membership almost vanished at high levels of knowledge. Panel A (left side) also suggests that knowledge seems to increase COVID-19 threat in the non-risk group, but this is not the case in the risk group, where the threat level remains high.

**Table 3:** Mediator variable models of the conditional mediation model (*N* = 4,096)^a^.

	**Model 1: Risk of exposure**			**Model 2: Risk of severe infection**		
	**Effect**	**SE**	**95%-CI**	**Effect**	**SE**	**95%-CI**
**Mediator variable models for the outcome COVID-threat**						
Risk group^b^	0.205***	0.048	[0.110, 0.300]	0.144**	0.046	[0.054, 0.233]
COVID knowledge	0.148***	0.032	[0.086, 0.211]	0.121***	0.033	[0.055, 0.186]
Risk group* COVID knowledge	−0.169**	0.054	[−0.274, −0.063]	−0.008	0.050	[−0.107, 0.090]
Constant	0.297***	0.030	[0.238, 0.357]	0.349***	0.032	[0.287, 0.412]
R^2^ (F-Test)	0.050 (44.980***)			0.096 (95.763***)		
**Conditional effect of risk group at different values of COVID knowledge**						
Low COVID knowledge	0.079***	0.012	[0.055, 0.102]	0.137***	0.011	[0.116, 0.159]
Medium COVID knowledge	0.053***	0.009	[0.035, 0.072]	0.136***	0.008	[0.120, 0.153]
High COVID knowledge	0.036**	0.011	[0.014, 0.058]	0.135***	0.010	[0.116, 0.155]

a *Medium COVID knowledge is indicated by the mean, while low knowledge is one standard deviation below the mean, and high knowledge is the maximum value 1 (because 1 standard deviation above the mean is above the maximum observed value in the data)*;

b *The respective risk group is indicated next to the model number; CI, 95% confidence interval; SE, Standard error; models controlled for sex and age*.

* *p < 0.05*,

** *p < 0.01*,

*** *p < 0.001*.

**Figure 3:**
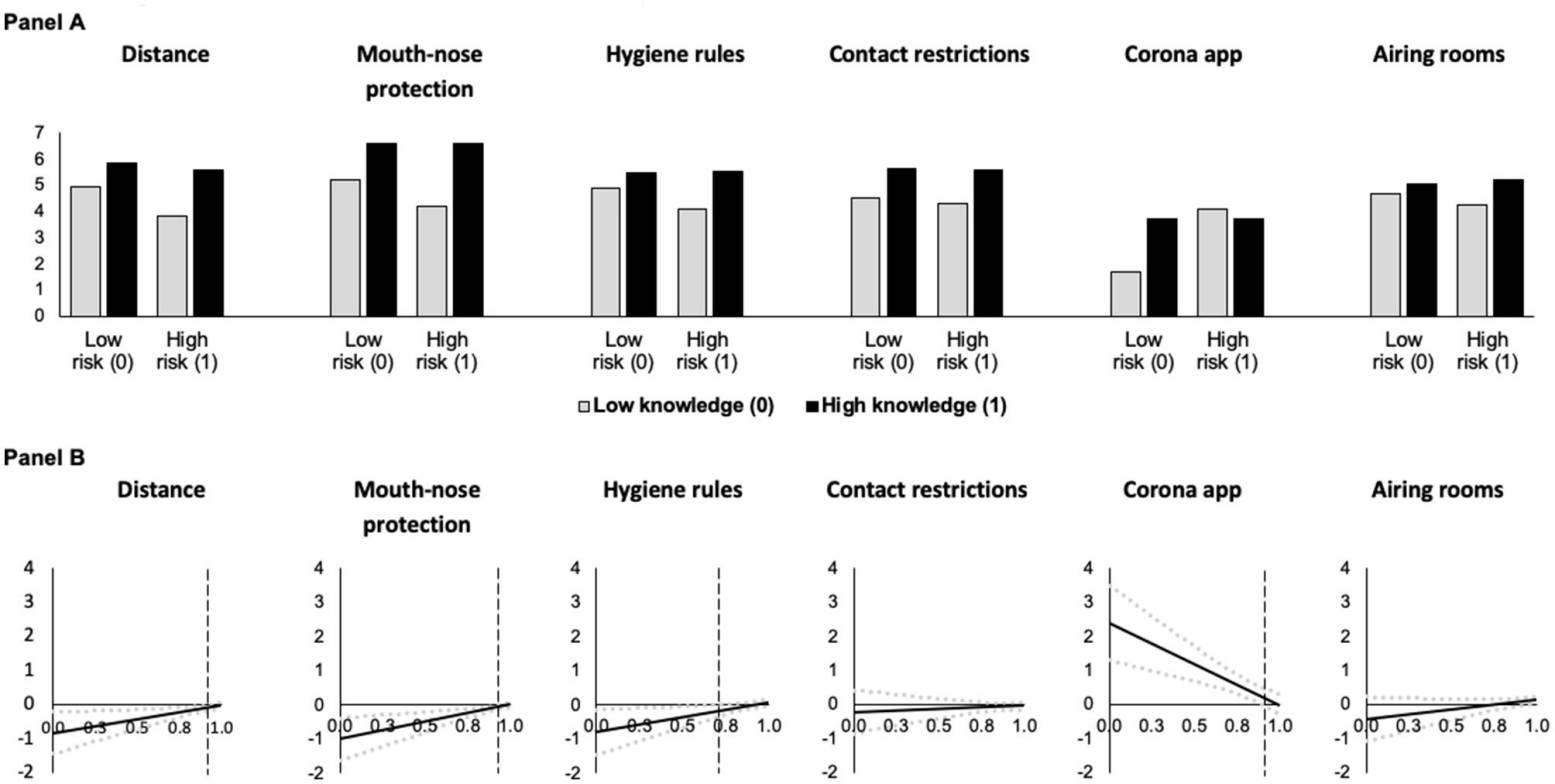
Predicted adherence as a function of risk of exposure at different levels of knowledge about COVID-19 **(A)** and conditional effect of risk of exposure as a function of knowledge about COVID-19 **(B)** (based on Models 3 and 8 in Tables 4, 5, *N* = 4, 096). In **(A)**, predictions are for men who are 18 years old who have medium levels of threat. In **(B)**, the black solid lines (**—**) indicate the conditional effect of risk group membership from the lowest (0) to the highest (1) level of knowledge about COVID-19 and dotted gray lines (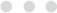) indicate the upper and lower 95% confidence interval. Vertical dashed black lines indicate the Johnson-Neyman value of knowledge above which the effect of risk group membership becomes statistically insignificant (*p* > 0.05). For the contact restrictions and airing rooms models, the effect is statistically insignificant throughout.

The dependent variable models (Tables 4, 5) test the conditional effect of risk of exposure on the adherence to behavioral measures via COVID-19 threat. At low levels of threat and knowledge, being at risk of exposure leads to lower levels of adherence to distance (Model 3), mouth-nose protection (Model 4), and hygiene rules (Model 5), as indicated by the statistically significant conditional main effects of risk of exposure (keeping in mind that these models control for the mediator COVID-19 threat). Furthermore, the statistically significant positive interaction effects between risk of exposure and COVID-knowledge in Models 3–5 suggest that the direct effect of belonging to this risk group on adherence to distance, mouth-nose protection, and hygiene rules becomes less negative with increasing knowledge. That is, at higher levels of knowledge, risk of exposure has a stronger positive effect on adherence and the negative conditional effect of risk of exposure vanishes (see Figure 3B).

**Table 4:** Dependent variable model of the conditional mediation model (*N* = 4,096)^a^.

	**Model 3: Distance**			**Model 4: Mouth-nose protection**			**Model 5: Hygiene rules**		
	***Effect***	***SE***	***95%-CI***	***Effect***	***SE***	***95%-CI***	***Effect***	***SE***	***95%-CI***
**Dependent variable model**									
Risk of exposure	−0.828**	0.313	[−1.442, −0.214]	−1.009**	0.316	[−1.628, −0.389]	−0.808*	0.338	[−1.470, −0.146]
COVID threat	4.139***	0.712	[2.743, 5.535]	4.369***	0.711	[2.976, 5.762]	3.616***	0.784	[2.078, 5.154]
COVID knowledge	2.792***	0.526	[1.761, 3.823]	3.586***	0.522	[2.562, 4.610]	1.592**	0.584	[0.446, 2.737]
Risk group*COVID knowledge	0.804*	0.336	[0.145, 1.463]	1.012**	0.337	[0.352, 1.672]	0.891*	0.367	[0.172, 1.611]
COVID threat*COVID knowledge	−3.223***	0.767	[−4.727, −1.718]	−3.897***	0.757	[−5.382, −2.413]	−1.807*	0.856	[−3.485, −0.129]
Constant	2.292***	0.486	[1.338, 3.246]	2.620***	0.488	[1.663, 3.578]	2.545***	0.533	[1.501, 3.590]
**Conditional direct effects of risk of exposure**									
Low COVID knowledge	−0.227**	0.071	[−0.366, −0.087]	−0.252***	0.070	[−0.390, −0.115]	−0.142	0.078	[−0.295, 0.011]
Medium COVID knowledge	−0.107**	0.041	[−0.187, −0.027]	−0.101**	0.034	[−0.168, −0.035]	−0.009	0.051	[−0.108, 0.091]
High COVID knowledge	−0.024	0.047	[−0.117, 0.069]	0.003	0.038	[−0.072, 0.078]	0.083	0.060	[−0.035, 0.202]
**Conditional direct effects of COVID threat**									
Low COVID knowledge	1.729***	0.155	[1.424, 2.034]	1.454***	0.157	[1.147, 1.762]	2.265***	0.175	[1.922, 2.607]
Medium COVID knowledge	1.249***	0.082	[1.089, 1.409]	0.874***	0.074	[0.728, 1.019]	1.995***	0.109	[1.782, 2.209]
High COVID knowledge	0.916***	0.099	[0.721, 1.111]	0.472***	0.085	[0.306, 0.638]	1.809***	0.134	[1.546, 2.072]
R^2^ (F–Test)	0.201 (94.427***)			0.193 (57.628***)			0.197 (109.252***)		
	***Effect***	***SE (Boot)***	***95%-CI*** ***(Boot)***	***Effect***	***SE (Boot)***	***95%-CI*** ***(Boot)***	***Effect***	***SE (Boot)***	***95%-CI*** ***(Boot)***
**Conditional indirect effects of risk of exposure via COVID threat**									
Low COVID knowledge	0.136	0.025	[0.090, 0.188]	0.114	0.022	[0.074, 0.161]	0.178	0.030	[0.121, 0.241]
Medium COVID knowledge	0.067	0.013	[0.042, 0.093]	0.047	0.009	[0.030, 0.066]	0.107	0.020	[0.068, 0.147]
High COVID knowledge	0.033	0.011	[0.012, 0.056]	0.017	0.006	[0.006, 0.031]	0.065	0.021	[0.024, 0.109]
	***Contrast***	***SE (Boot)***	***95%-CI*** ***(Boot)***	***Contrast***	***SE (Boot)***	***95%-CI*** ***(Boot)***	***Contrast***	***SE (Boot)***	***95%-CI*** ***(Boot)***
**Pairwise contrasts between conditional indirect effects of risk of exposure via COVID threat**									
Medium vs. low COVID knowledge	−0.069	0.018	[−0.107, −0.036]	−0.068	0.017	[−0.102, −0.037]	−0.071	0.021	[−0.115, −0.031]
High vs. low COVID knowledge	−0.103	0.025	[−0.154, −0.056]	−0.097	0.022	[−0.143, −0.056]	−0.113	0.032	[−0.177, −0.051]
High vs. medium COVID knowledge	−0.034	0.007	[−0.048, −0.020]	−0.030	0.006	[−0.042, −0.019]	−0.041	0.011	[−0.063, −0.020]

* *p < 0.05*,

** *p < 0.01*,

*** *p < 0.001*.

a *Medium COVID knowledge is indicated by the mean, while low knowledge is one standard deviation below the mean, and high knowledge is the maximum value 1 (because 1 standard deviation above the mean is above the maximum observed value in the data); CI, 95% confidence interval; SE, Standard error; Boot, Bootstrap sample size = 10,000. Models controlled for sex and age*.

**Table 5:** Dependent variable model of the conditional mediation model (*N* = 4,096)^a^.

	**Model 6: Contact restrictions**			**Model 7: Corona app**			**Model 8: Airing rooms**		
	***Effect***	***SE***	***95%–CI***	***Effect***	***SE***	***95%-CI***	***Effect***	***SE***	***95%-CI***
**Dependent variable model**									
Risk of exposure	−0.211	0.323	[−0.844, 0.421]	2.391***	0.560	[1.293, 3.489]	−0.426	0.334	[−1.081, 0.230]
COVID threat	4.992***	0.776	[3.470, 6.513]	2.892**	0.952	[1.026, 4.758]	4.264***	0.781	[2.732, 5.796]
COVID knowledge	2.994***	0.573	[1.872, 4.117]	2.009***	0.550	[0.930, 3.087]	1.899***	0.563	[0.795, 3.003]
Risk group*COVID knowledge	0.177	0.352	[−0.513, 0.867]	−2.393***	0.635	[−3.639, −1.148]	0.553	0.366	[−0.164, 1.269]
COVID threat*COVID knowledge	−3.277***	0.842	[−4.928, −1.626]	0.076	1.072	[−2.026, 2.177]	−2.664**	0.850	[−4.331, −0.997]
Constant	1.347*	0.524	[0.320, 2.375]	0.177	0.489	[−0.783, 1.136]	1.898***	0.513	[0.891, 2.905]
**Conditional direct effects of risk of exposure**									
Low COVID knowledge	−0.079	0.074	[−0.225, 0.067]	0.601***	0.144	[0.319, 0.884]	−0.012	0.080	[−0.170, 0.145]
Medium COVID knowledge	−0.052	0.050	[−0.150, 0.045]	0.245	0.128	[−0.006, 0.496]	0.070	0.058	[−0.043, 0.183]
High COVID knowledge	−0.034	0.060	[−0.151, 0.083]	−0.002	0.154	[−0.305, 0.301]	0.127	0.068	[−0.006, 0.261]
**Conditional direct effects of COVID threat**									
Low COVID knowledge	2.541***	0.175	[2.197, 2.885]	2.949***	0.229	[2.501, 3.397]	2.272***	0.182	[1.915, 2.269]
Medium COVID knowledge	2.053***	0.108	[1.842, 2.264]	2.960***	0.189	[2.590, 3.330]	1.875***	0.121	[1.637, 2.113]
High COVID knowledge	1.715***	0.130	[1.461, 1.969]	2.968***	0.233	[2.512, 3.424]	1.600***	0.144	[1.318, 1.883]
R^2^ (F-Test)	0.219 (112.318***)			0.064 (52.652***)			0.151 (87.671***)		
	***Effect***	***SE (Boot)***	***95%-CI*** ***(Boot)***	***Effect***	***SE (Boot)***	***95%-CI*** ***(Boot)***	***Effect***	***SE (Boot)***	***95%-CI*** ***(Boot)***
**Conditional indirect effects of risk of exposure via COVID threat**									
Low COVID knowledge	0.200	0.034	[0.136, 0.269]	0.232	0.039	[0.158, 0.313]	0.179	0.032	[0.119, 0.244]
Medium COVID knowledge	0.110	0.020	[0.071, 0.151]	0.158	0.030	[0.102, 0.218]	0.100	0.019	[0.064, 0.139]
High COVID knowledge	0.062	0.020	[0.024, 0.102]	0.107	0.035	[0.041, 0.178]	0.058	0.019	[0.022, 0.096]
	***Contrast***	***SE (Boot)***	***95%-CI*** ***(Boot)***	***Contrast***	***SE (Boot)***	***95%-CI*** ***(Boot)***	***Contrast***	***SE (Boot)***	***95%-CI*** ***(Boot)***
**Pairwise contrasts between conditional indirect effects of risk of exposure via COVID threat**									
Medium vs. low COVID knowledge	−0.090	0.024	[−0.138, −0.046]	−0.074	0.027	[−0.129, −0.022]	−0.078	0.022	[−0.124, −0.037]
High vs. low COVID knowledge	−0.138	0.034	[−0.207, −0.072]	−0.125	0.044	[−0.212, −0.040]	−0.121	0.033	[−0.187, −0.059]
High vs. medium COVID knowledge	−0.048	0.011	[−0.069, −0.027]	−0.051	0.017	[−0.085, −0.017]	−0.043	0.010	[−0.063, −0.022]

* *p < 0.05*,

** *p < 0.01*,

*** *p < 0.001.*

a *Medium COVID knowledge is indicated by the mean, while low knowledge is one standard deviation below the mean, and high knowledge is the maximum value 1 (because 1 standard deviation above the mean is above the maximum observed value in the data); CI, 95% confidence interval; SE, Standard error; Boot, Bootstrap sample size = 10,000. Models controlled for sex and age*.

Risk of exposure has neither statistically significant conditional main effects on adherence to contact restrictions (Model 6) and airing rooms (Model 8), nor statistically significant interaction effects between risk of exposure and knowledge in these models. Under the model conditions, we observed a statistically significant positive conditional main effect of risk of exposure on use of a corona app (Model 7) when COVID-19 threat and knowledge were low. For this behavior, we also found a statistically significant negative interaction effect between risk of exposure and knowledge. The findings and visualizations in Figures 3A,B suggest that if knowledge is high, being in a risk group has no direct effect, while at low levels of knowledge, risk group membership leads to more use of a corona app.

**Figure 4:**
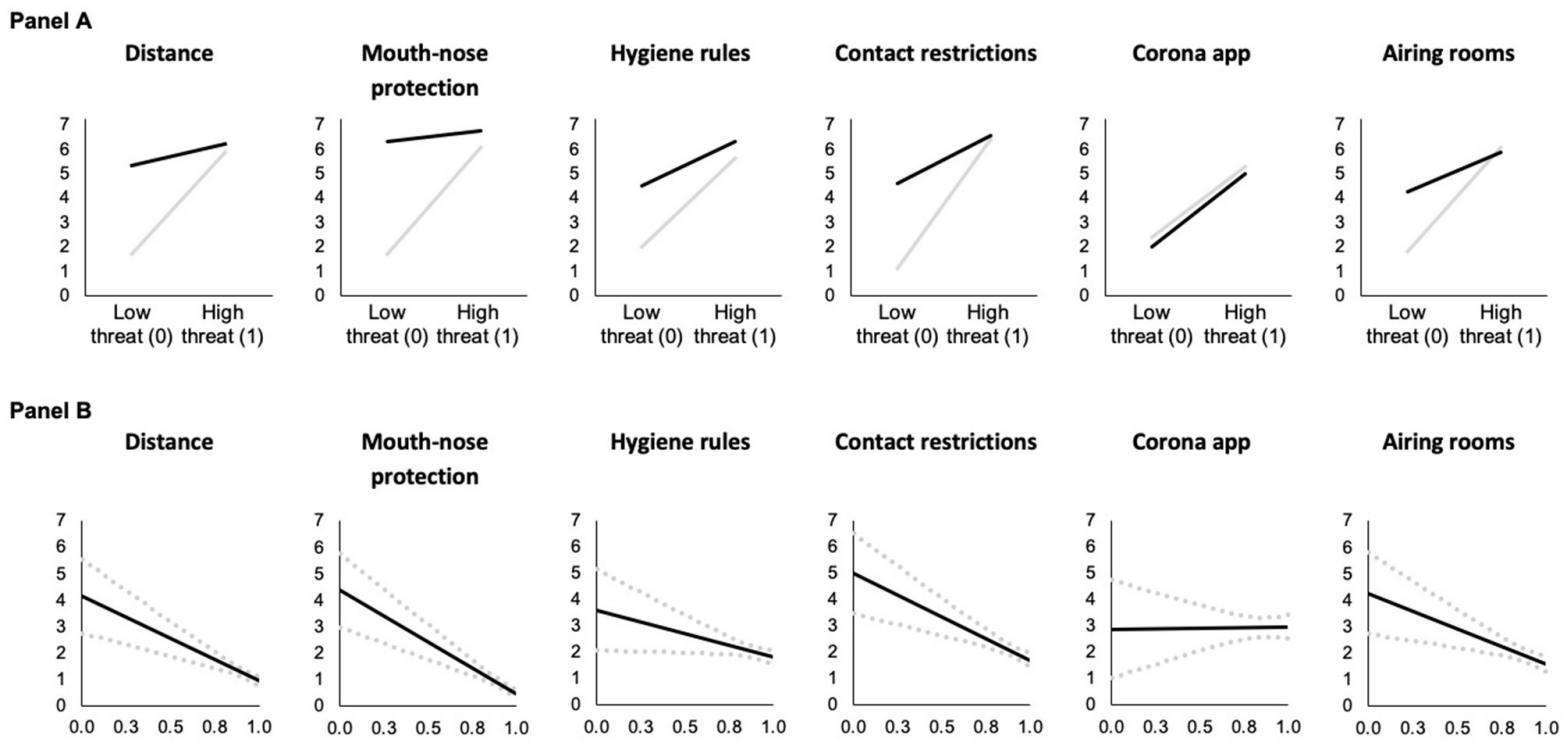
Predicted adherence as a function of COVID-19 threat at different levels of knowledge about COVID-19 **(A)** and conditional effect of COVID-19 threat as a function of knowledge about COVID-19 in the risk of exposure models **(B)** (based on Models 3 and 8 in Tables 4, 5, *N* = 4, 096). In **(A)**, gray lines (**—**) indicate low knowledge (0) and black lines (**—**) indicate high knowledge (1). Predictions are for men who are 18 years and at risk of exposure. In **(B)**, the black solid lines (**—**) indicate the conditional effect perceived COVID-19 threat from the lowest (0) to the highest (1) level of knowledge about COVID-19 and dotted gray lines (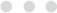) indicate the upper and lower 95% confidence interval; and all Johnson-Neyman values are statistically significant (*p* < 0.05).

In all models, the mediator COVID-19 threat has a statistically significant positive conditional direct main effect on adherence to each of the COVID-19 behavioral measures, i.e., adherence increases as threat increases for individuals with low knowledge (Figures 4A,B). This positive effect becomes smaller with increasing COVID knowledge as indicated by the statistically significant negative interaction effects between COVID-19 threat and COVID knowledge (with the exception of Model 7 for the use of a corona app, where threat has a comparable effect across all levels of knowledge).

We also found positive indirect effects of risk of exposure via COVID-19 threat on all adherence measures (confidence intervals do not include zero). These indirect effects become weaker at higher levels of knowledge (as indicated by the conditional indirect effects and the pairwise contrasts), showing support of moderated mediation effects.

### Risk of Severe COVID-19 Infection

The mediator variable model in Model 2 (Table 3) tests the effects of risk of severe COVID-19 infection and knowledge about COVID-19 on COVID-19 threat. Results indicate that individuals at risk of severe COVID-19 infection with low knowledge and non-risk group members with more COVID-knowledge have increased COVID-19 threat (indicated by the statistically significant conditional main effects of risk group and knowledge). The effect of risk of severe infection is not moderated by COVID-knowledge (Model 2). See a visualization of the effects in Figures 2A,C.

**Figure 5:**
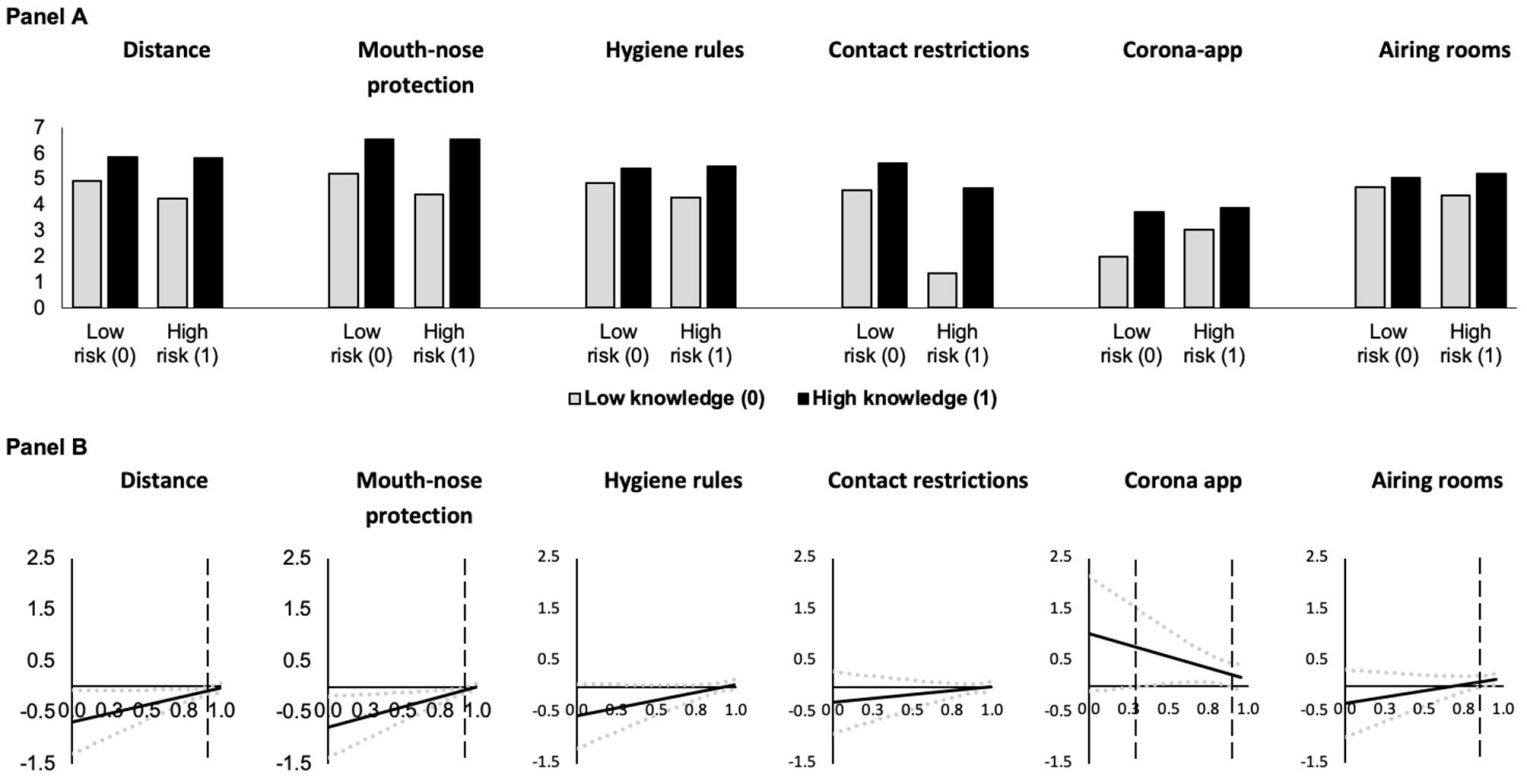
Predicted adherence as a function of risk of severe infection at different levels of knowledge about COVID-19 **(A)** and conditional effect of risk of severe infection as a function of knowledge about COVID-19 **(B)** (based on Models 9 and 14 in Tables 6, 7, *N* = 4, 096). In **(A)**, predictions are for men who are 18 years old who have medium levels of threat. In **(B)**, the black solid lines (**—**) indicate the conditional effect of risk group membership from the lowest (0) to the highest (1) level of knowledge about COVID-19 and dotted gray lines (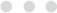) indicate the upper and lower 95% confidence interval. Vertical dashed black lines indicate the Johnson-Neyman value of knowledge above which the effect of risk group membership becomes statistically insignificant (*p* > 0.05). For the corona app use model, the effect of knowledge is statistically significant between the vertical dashed lines, while the effect is statistically insignificant throughout the hygiene rules and contact restrictions models.

The dependent variable models (Tables 6, 7) test the conditional effect of risk of severe infection on adherence to behavioral measures via COVID-19 threat. The models show that risk of severe infection has a statistically significant conditional negative main effect on adherence to distance (Model 9) and mouth-nose protection measures (Model 10) when COVID-19 threat and COVID-knowledge are lowest and the mediator, COVID-19 threat, is held constant. Thus, risk group membership decreases adherence to these recommendations under these conditions. The statistically significant positive interaction effects between risk of severe infection and COVID-knowledge suggest that the negative effects on adherence to distance and mouth-nose protection become less negative with increasing knowledge. The visual description shows that at high levels of knowledge, the negative effect of risk group membership on adherence to these measures becomes nonexistent (Figure 5B). The conditional main effects of risk group membership are statistically insignificant for the other behaviors (Models 11 to 14, see also Figures 5A,B). Although the interaction effect between risk group and knowledge was statistically insignificant in Model 14, the Johnson-Neyman technique showed that knowledge could moderate the relation between risk group and knowledge in the airing rooms model when knowledge was high (i.e., above 0.88). Also, knowledge about COVID-19 played a moderating role in the use of a corona app model when knowledge was between 0.300 and 0.891. The findings and the visualizations in Figures 5A,B suggest that at higher levels of knowledge, being in a risk group has no direct effect, while at lower levels of knowledge, risk group membership is associated with more frequent use of a corona app.

**Table 6:** Dependent variable model of the conditional mediation model (*N* = 4,096)^a^.

	**Model 9: Distance**			**Model 10: Mouth-nose protection**			**Model 11: Hygiene rules**		
	***Effect***	***SE***	***95%-CI***	***Effect***	***SE***	***95%-CI***	***Effect***	***SE***	***95%-CI***
**Dependent variable model**									
Risk of severe infection	−0.693*	0.316	[−1.314, −0.073]	−0.782*	0.303	[−1.377, −0.187]	−0.572	0.319	[−1.197, 0.052]
COVID threat	4.234***	0.732	[2.799, 5.668]	4.463***	0.722	[3.048, 5.878]	3.686***	0.806	[2.106, 5.266]
COVID knowledge	2.819***	0.518	[1.803, 3.834]	3.632***	0.516	[2.621, 4.643]	1.654**	0.579	[0.519, 2.789]
Risk group*COVID knowledge	0.676*	0.341	[0.008, 1.344]	0.779*	0.324	[0.144, 1.414]	0.623	0.345	[−0.054, 1.299]
COVID threat*COVID knowledge	−3.312***	0.790	[−4.862, −1.763]	−3.989***	0.770	[−5.499, −2.480]	−1.890*	0.881	[−3.617, −0.164]
Constant	2.231***	0.478	[1.294, 3.168]	2.543***	0.482	[1.599, 3.487]	2.487***	0.527	[1.454, 3.520]
**Conditional direct effects of risk of severe infection**									
Low COVID knowledge	−0.188**	0.070	[−0.324, −0.051]	−0.199**	0.066	[−0.329, −0.069]	−0.107	0.073	[−0.249, 0.035]
Medium COVID knowledge	−0.087*	0.037	[−0.160, −0.013]	−0.083**	0.031	[−0.144, −0.022]	−0.014	0.045	[−0.101, 0.073]
High COVID knowledge	−0.017	0.045	[−0.105, 0.071]	−0.003	0.036	[−0.074, 0.068]	0.050	0.053	[−0.054, 0.154]
**Conditional direct effects of COVID threat**									
Low COVID knowledge	1.756***	0.159	[1.445, 2.068]	1.479***	0.159	[1.167, 1.791]	2.272***	0.178	[1.923, 2.621]
Medium COVID knowledge	1.263***	0.084	[1.099, 1.427]	0.885***	0.076	[0.736, 1.034]	1.990***	0.111	[1.773, 2.207]
High COVID knowledge	0.921***	0.103	[0.719, 1.124]	0.473***	0.088	[0.302, 0.645]	1.795***	0.138	[1.525, 2.066]
R^2^ (F–Test)	0.200 (93.197***)			0.191 (57.307***)			0.196 (109.987***)		
	***Effect***	***SE (Boot)***	***95%-CI*** ***(Boot)***	***Effect***	***SE (Boot)***	***95%-CI*** ***(Boot)***	***Effect***	***SE (Boot)***	***95%-CI*** ***(Boot)***
**Conditional indirect effects of risk of severe infection via COVID threat**									
Low COVID knowledge	0.241	0.030	[0.186, 0.303]	0.203	0.028	[0.152, 0.261]	0.312	0.035	[0.247, 0.384]
Medium COVID knowledge	0.172	0.016	[0.142, 0.204]	0.120	0.013	[0.097, 0.147]	0.271	0.023	[0.227, 0.318]
High COVID knowledge	0.125	0.017	[0.093, 0.159]	0.064	0.013	[0.040, 0.090]	0.243	0.026	[0.193, 0.296]
	***Contrast***	***SE (Boot)***	***95%-CI*** ***(Boot)***	***Contrast***	***SE (Boot)***	***95%-CI*** ***(Boot)***	***Contrast***	***SE (Boot)***	***95%-CI*** ***(Boot)***
**Pairwise contrasts between conditional indirect effects of risk of severe infection via COVID threat**									
Medium vs. low COVID knowledge	−0.069	0.022	[−0.113, −0.028]	−0.083	0.020	[−0.125, −0.045]	−0.041	0.024	[−0.091, 0.005]
High vs. low COVID knowledge	−0.117	0.034	[−0.184, −0.051]	−0.139	0.031	[−0.204, −0.080]	−0.069	0.039	[−0.149, 0.006]
High vs. medium COVID knowledge	−0.047	0.013	[−0.072, −0.022]	−0.056	0.012	[−0.080, −0.034]	−0.028	0.015	[−0.058, 0.002]

* *p < 0.05*,

** *p < 0.01*,

*** *p < 0.001.*

a *Medium COVID knowledge is indicated by the mean, while low knowledge is one standard deviation below the mean, and high knowledge is the maximum value 1 (because 1 standard deviation above the mean is above the maximum observed value in the data); CI, 95% confidence interval; SE, Standard error; Boot, Bootstrap sample size = 10,000. Models controlled for sex and age*.

**Table 7:** Dependent variable model of the conditional mediation model (*N* = 4,096)^a^.

	**Model 12: Contact restrictions**			**Model 13: Corona app**			**Model 14: Airing rooms**		
	***Effect***	***SE***	***95%-CI***	***Effect***	***SE***	***95%-CI***	***Effect***	***SE***	***95%-CI***
**Dependent variable model**									
Risk of severe infection	−0.312	0.305	[−0.909, 0.285]	1.023	0.573	[−0.100, 2.145]	−0.338	0.333	[−0.992, 0.315]
COVID threat	5.074***	0.793	[3.520, 6.629]	3.069**	0.987	[1.134, 5.003]	4.348***	0.797	[2.787, 5.910]
COVID knowledge	2.984***	0.569	[1.869, 4.099]	1.830**	0.558	[0.735, 2.925]	1.959***	0.558	[0.865, 3.054]
Risk group*COVID knowledge	0.315	0.332	[−0.335, 0.966]	−0.888	0.646	[−2.154, 0.377]	0.504	0.366	[−0.214, 1.222]
COVID threat*COVID knowledge	−3.365***	0.863	[−5.057, −1.673]	−0.163	1.112	[−2.343, 2.018]	−2.809**	0.870	[−4.514, −1.103]
Constant	1.344*	0.519	[0.326, 2.362]	0.448	0.498	[−0.529, 1.425]	1.892***	0.508	[0.896, 2.888]
**Conditional direct effects of risk of severe infection**									
Low COVID knowledge	−0.076	0.069	[−0.211, 0.060]	0.358*	0.140	[0.083, 0.634]	0.039	0.077	[−0.111, 0.190]
Medium COVID knowledge	−0.029	0.044	[−0.116, 0.058]	0.226	0.119	[−0.006, 0.458]	0.114*	0.053	[0.010, 0.219]
High COVID knowledge	0.004	0.054	[−0.101, 0.109]	0.134	0.145	[−0.150, 0.418]	0.166*	0.065	[0.039, 0.294]
**Conditional direct effects of COVID threat**									
Low COVID knowledge	2.558***	0.178	[2.209, 2.906]	2.947***	0.236	[2.485, 3.409]	2.248***	0.184	[1.886, 2.610]
Medium COVID knowledge	2.056***	0.111	[1.840, 2.273]	2.923***	0.195	[2.541, 3.305]	1.830***	0.125	[1.585, 2.074]
High COVID knowledge	1.709***	0.135	[1.445, 1.973]	2.906***	0.241	[2.434, 3.378]	1.540***	0.150	[1.247, 1.833]
R^2^ (F–Test)	0.219 (113.256***)			0.062 (49.866***)			0.152 (90.340***)		
	***Effect***	***SE (Boot)***	***95%-CI*** ***(Boot)***	***Effect***	***SE (Boot)***	***95%-CI*** ***(Boot)***	***Effect***	***SE (Boot)***	***95%-CI*** ***(Boot)***
**Conditional indirect effects of risk of severe infection via COVID threat**									
Low COVID knowledge	0.351	0.039	[0.278, 0.430]	0.405	0.047	[0.318, 0.501]	0.309	0.037	[0.239, 0.386]
Medium COVID knowledge	0.280	0.023	[0.236, 0.327]	0.398	0.037	[0.328, 0.473]	0.249	0.024	[0.205, 0.297]
High COVID knowledge	0.231	0.025	[0.184, 0.282]	0.393	0.044	[0.310, 0.484]	0.208	0.026	[0.160, 0.261]
	***Contrast***	***SE (Boot)***	***95%-CI*** ***(Boot)***	***Contrast***	***SE (Boot)***	***95%-CI*** ***(Boot)***	***Contrast***	***SE (Boot)***	***95%-CI*** ***(Boot)***
**Pairwise contrasts between conditional indirect effects of risk of severe infection via COVID threat**									
Medium vs. low COVID knowledge	−0.071	0.027	[−0.126, −0.021]	−0.007	0.032	[−0.072, 0.053]	−0.060	0.025	[−0.111, −0.012]
High vs. low COVID knowledge	−0.120	0.042	[−0.204, −0.039]	−0.012	0.053	[−0.118, 0.092]	−0.101	0.040	[−0.179, −0.023]
High vs. medium COVID knowledge	−0.049	0.016	[−0.079, −0.018]	−0.005	0.022	[−0.047, 0.038]	−0.041	0.015	[−0.070, −0.011]

* *p < 0.05*,

** *p < 0.01*,

*** *p < 0.001.*

a *Medium COVID knowledge is indicated by the mean, while low knowledge is one standard deviation below the mean, and high knowledge is the maximum value 1 (because 1 standard deviation above the mean is above the maximum observed value in the data); CI, 95% confidence interval; SE, Standard error; Boot, Bootstrap sample size = 10,000. Models controlled for sex and age*.

**Figure 6:**
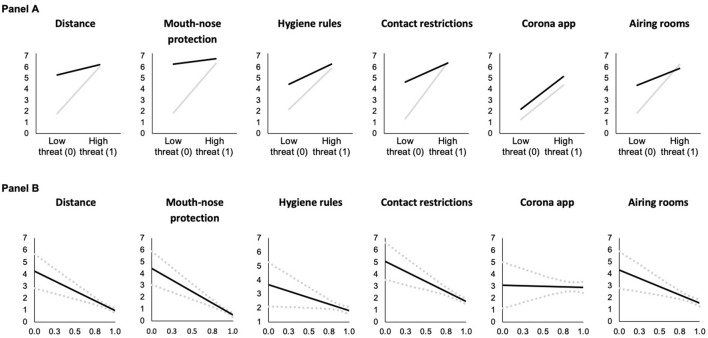
Predicted adherence as a function of COVID-19 threat at different levels of knowledge about COVID-19 **(A)** and conditional effect of COVID-19 threat as a function of knowledge about COVID-19 **(B)** in risk of severe infection models (based on Models 9 and 14 in Tables 6, 7, *N* = 4, 096). In **(A)**, gray lines (**—**) indicate low knowledge (0) and black lines (**—**) indicate high knowledge (1). Predictions are for men who are 18 years old and at risk of severe COVID-19 infection. In **(B)**, the black solid lines (**—**) indicate the conditional effect of perceived COVID-19 threat from the lowest (0) to the highest (1) level of knowledge about COVID-19 and dotted gray lines (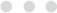) indicate the upper and lower 95% confidence interval; and all Johnson-Neyman values are statistically significant (*p* < 0.05).

Also, in these models the mediator COVID-19 threat has a statistically significant positive conditional main effect on adherence to COVID-19 behavioral measures (Figures 6A,B). Thus, holding risk of severe infection constant, COVID-19 threat increases adherence at low levels of knowledge. This positive effect decreases with increasing COVID knowledge (Figures 6A,B) as indicated by the statistically significant negative interaction effects between COVID-19 threat and COVID knowledge (with the exception of Model 13 for the use of a corona app).

Again, we found positive indirect effects of risk of severe infection via COVID-19 threat on adherence (confidence intervals do not include zero), but these indirect effects become weaker at higher levels of knowledge (as indicated by the conditional indirect effects and the pairwise contrasts), demonstrating moderated mediation effects.

## Discussion

Given the need to better understand the conditions under which individuals engage in behavior that prevents the spread of COVID-19, this study employs a representative sample of the adult population in Germany to examine the relationship between risk group membership and adherence to six COVID-19 behavioral measures recommended by the central disease monitoring and prevention institution of the federal German government. We tested whether this relationship is mediated by perceived threat of COVID-19 and moderated by knowledge about COVID-19. The two risk groups examined are individuals who perceive themselves at higher risk of exposure to COVID-19 (e.g., essential workers in hospitals or schools) and of severe COVID-19 infection (e.g., suffering from diabetes or a lung or heart disease).

### Perceived Threat as a Mediator Between Risk Group and Adherence

We found that more individuals perceived themselves as at higher risk of severe infection than exposure to the virus. Membership to either of the risk groups (risk of exposure or risk of severe infection) was indirectly related to increased adherence to all six behavioral measures via elevated COVID-19 threat across different levels of knowledge about COVID-19. Previously, associations have typically been examined separately between risk group membership and adherence (12, 15, 16, 43), risk (objective and subjective) and affective reactions such as anxiety, fear, or worry (18–20, 22, 23), as well as between such affective reactions and adherence to behavioral measures (14, 23, 25). Similar to initial research with a Chinese sample (26), our results show that COVID-19 threat mediates the effect of both risk group membership indicators on the examined preventive behaviors. This finding also corresponds to other studies on health decision making, which find a mediating effect of worry explaining the relationship between perceived risk and health behavior (44–46). Our results, in conjunction with previous literature on the role of affect in health decision making, suggest that one mechanism explaining of the increased adherence by risk group members is that they have more feelings of anxiety, fear, and worry related to COVID-19. Furthermore, our findings support the theorized role of anxiety as an adaptive emotion to help individuals detect and protect themselves from threats (24), as well as the theorized benefit of worrying to help maintain awareness about a potential threat (47). While theories about health decision making tend to focus on cognitive appraisals, the mediation role of threat explaining differences in adherence between risk group members and non-risk group members underlines the usefulness of incorporating affective reactions into health behavior theories (46). Although we found indirect effects of risk group membership via COVID-19 threat on adherence at different levels of knowledge about COVID-19, this indirect effect decreased with knowledge. Therefore, in the following, we will shed light on how each of the individual pathways (Figure 1) were moderated by knowledge.

### Knowledge as a Moderator of the Relation Between Risk Group and Perceived Threat

While individuals at risk of severe infection experience more COVID-19 threat independent of their knowledge about COVID-19, such knowledge negatively moderated the positive association between being at risk of exposure and COVID-19 threat. Hence, the effect of being at risk of exposure on COVID-19 threat was greatest when knowledge was low, it decreased as knowledge increased, and almost vanished at high levels of knowledge. Although we found a negative interaction effect, the results do not suggest that knowledge buffers the effect of being at risk of exposure, but rather it increases COVID-19 threat for individuals not at such risk. This conditional effect of risk group membership expands upon the literature on the associations between COVID-19 risk and threat-related emotions (18–20, 22, 23) and also adds to the larger literature on the relationship between health-related risk perceptions and worry [see, for example, cancer-related studies (48–50)]. For individuals not at risk of exposure, increased knowledge increases the perceived threat. This may be due to a better understanding of the virus, resulting in the perception that these individuals also can be exposed to COVID-19, even if they are not at particularly high risk of having contact with others (e.g., due to their job).

### Knowledge as a Moderator of the Relation Between Perceived Threat and Adherence

Adding to previous findings on the positive relationship between threat-related emotions and adherence to behavioral measures against the spread of COVID-19 (14, 23, 25), we found that COVID-19 threat has a positive conditional main effect on adherence to all six behavioral measures examined. Thus, there is a positive association between threat and adherence at low levels of knowledge when holding risk group membership constant. However, this association decreased as knowledge increased (in all models except use of a corona app). This finding is similar to Jørgensen et al. (14) who found that self-efficacy negatively moderates the positive effect of worry on adherence to behavioral measures against COVID-19. One interpretation is that adherence is generally higher if perceived COVID-19 threat is high and additional knowledge cannot further increase adherence. This can be understood as a ceiling effect. On the other hand, if perceived COVID-19 threat is low, knowledge has greater potential to increase adherence. This identifies increasing knowledge as a path to adherence with less fear.

### Knowledge as a Moderator of the Relation Between Risk Group and Adherence

Moreover, our analysis also suggests that – net of the mediator COVID-19 threat – risk group membership had remaining conditional negative effects on several adherence measures (e.g., distancing and wearing mouth-nose protection) depending on knowledge about COVID-19. Hence, risk group members had reduced adherence to some behaviors (i.e., distance, mouth-nose protection, and hygiene rules) at low levels of knowledge. However, this effect tended to vanish as knowledge increased. This suggests that individuals who are at risk and less knowledgeable about the virus seem to ignore the behavioral measures to a larger degree and, thereby, put themselves and others at higher risk of infection and severe outcomes. Future studies should elaborate further mechanisms to understand this remaining effect of risk group membership.

### Unique Patterns Observed for Use of a Corona app

Given the unique patterns observed for use of a corona app, we want to elaborate briefly on this aspect. Of the six behavioral measures examined, app use is the least adhered to behavior. One reason might be that, unlike the other behaviors examined, use of an app requires having a smartphone that is connected to the app store and voluntarily providing information about one's location, thus everyone may not be able to download the application nor willing to provide this information to the German government or other agencies. Furthermore, it is dissimilar to the other measures in that it is used to prevent the spread of COVID-19, rather than to protect oneself against infection. These differences between the measures correspond to the top three reasons for not using the application in the German population: “privacy concerns, doubts about the effectiveness of the app and lack of technical equipment” [(51), p.49].

### Strengths, Limitations, and Avenues for Future Research

The current study advances existing research on the relationship between risk group membership and adherence to all six recommended behavioral measures against the spread of COVID-19 as well as processes and conditions under which this relationship occurs, by examining the mediating and moderating roles of perceived COVID-19 threat and knowledge about COVID-19. It is one of the few representative studies which explores the relationship between increased risk and adherence and tests moderated mediation effects. Unlike many studies on adherence to behavioral measures against the spread of COVID-19 that examine only single behaviors or compound measures of several behaviors, this study examined adherence to each measure separately, thereby shedding light on the similarities and differences in which adherence to these measures occurs. We demonstrate that adherence to one measure, the use of a corona app, is partially distinct from adherence to the other recommended measures, thus informing future research that this behavior should be examined separately.

While we used two single item measures to assess self-perceived risk group membership, future studies may also measure objective risk group membership by providing respondents with lists of risk factors (e.g., for severe infection including diabetes, chronic lung diseases, obesity, high age, cortisone use, etc.) to test how these objective measures translate to self-perceived risk group membership or how particular co-morbidities affect perceived threat. We also invite future studies to examine the pathways identified with longitudinal or experimental studies and examine to which extent the variables investigated here can explain adherence in relation to other factors, such as depressive tendencies (52), trait anxiety (21), self-efficacy (14), and a desire to help protect others (53), as further confounders, mediators, or moderators.

To our knowledge, there were no validated measures of COVID-19 knowledge during the conceptualization of the study. Future studies can extend upon our work by validating the measure of COVID-19 knowledge developed for this study or using validated measures of knowledge about COVID-19.

Given that the number of COVID-19 cases and the filling of the intensive care units have varied over the course of the pandemic as well as across regions, future studies (both in- and outside the German context) would be helpful. Another opportunity for future studies is to test the model with other preventative COVID-19-related behavior, such as getting vaccinated and booster shots in a timely manner. Moreover, we used self-report measures of adherence and previous research suggests that people over-estimate their adherence with these measures (54). One reason could be social desired responding as a function of low perceptions of anonymity. In our study there were low levels of item-nonresponse (0.02 to 2.02% per item), what can be seen as another indicator for limited problems of social desirability.

## Conclusion

The present study demonstrates that individuals who perceive themselves as at higher risk of exposure to COVID-19 and of severe COVID-19 infection experience increased perceived COVID-19 threat, which entails worry, stress, and anxiety related to COVID-19. While extreme levels of anxiety can be detrimental to wellbeing (55), we find that perceived COVID-19 threat is a mechanism explaining the increased adherence to behavioral measures by risk group members. Individuals who perceive low levels of threat may less likely seek to protect themselves and, thus, have a higher risk of exposure to the virus. Therefore, moderate levels of threat-related emotions are not inherently maladaptive as they can lead to protective behavior in the context of the pandemic (21, 22, 25, 33). However, increasing the level of COVID-19 threat in the population with knowledge interventions could be potentially harmful for individuals with existing high levels of anxiety and lead to the development or worsening of mental health issues. Hence, knowledge about COVID-19 can be viewed as a double-edge sword as it increases both perceived threat and adherence.

## Data Availability Statement

The raw data supporting the conclusions of this article are available here: (56).

## Ethics Statement

This study involving human participants was reviewed and approved by Faculty of Management, Economics and Social Sciences at the University of Cologne (Ethics Approval Number: 200015DM_extension). The participants provided their written informed consent to participate in this study.

## Author Contributions

SS: conceptualization, methodology, investigation, statistical analysis, data curation, writing—original draft, visualization, project administration, and funding acquisition. ST: conceptualization, writing—original draft, and visualization. AE: conceptualization and writing—original draft. FH: conceptualization, investigation, and writing—original draft. All authors read and approved the final manuscript.

## Funding

This work was supported by the Dr. Hans Riegel Foundation [to SS].

## Conflict of Interest

The authors declare that the research was conducted in the absence of any commercial or financial relationships that could be construed as a potential conflict of interest.

## Publisher's Note

All claims expressed in this article are solely those of the authors and do not necessarily represent those of their affiliated organizations, or those of the publisher, the editors and the reviewers. Any product that may be evaluated in this article, or claim that may be made by its manufacturer, is not guaranteed or endorsed by the publisher.

## Footnotes

^1^In an elaborate scoring and control process, the panel is subjected to permanent quality control.

^2^In Germany, there are smartphone applications available to the public (e.g., Corona-Warn-App), which alert users if they were in contact with an individual who recently tested positive for COVID-19.

^3^With bootstrapping, the effects of variables are assessed in a way that maximizes power. It is robust against non-normality.

^4^High knowledge refers to the maximum value 1 (because 1 standard deviation above the mean is above the maximum observed value in the data).
