# Supplementary material for: A Moderated Mediation Model Explaining the Relationship Between Risk-Group Membership, Threat Perception, Knowledge, and Adherence to COVID-19 Behavioral Measures
Source: Front Public Health. 2022 May 19;10:842368. doi: 10.3389/fpubh.2022.842368 (PMC9160797; doi:10.3389/fpubh.2022.842368)
Supplement: Supplementary file 1 [file Table_1.DOCX]

Supplementary Material

# Supplementary Table

**Supplementary Table 1: Knowledge about COVID-19 Items.**

| **German (original language)** | | **English (translation)** | |
| --- | --- | --- | --- |
| 1. | Ist trockener Husten ein Symptom von Corona? | 1. | Is a dry cough a symptom of corona? |
| 2. | Schützt Alkoholkonsum vor Corona? | 2. | Does consuming alcohol protect against corona? |
| 3. | Wird bei der Einnahme einer hohen Dosis Vitamin C das Virus getötet? | 3. | Does taking a high dose of vitamin C kill the virus? |
| 4. | Haben Menschen mit Herz-Kreislauf-Erkrankungen ein erhöhtes Risiko für einen schweren Krankheitsverlauf von Corona? | 4. | Do people with cardiovascular disease have a higher risk of severe corona infection? |
| 5. | Sind Antibiotika wirksam bei der Behandlung von Corona? | 5. | Are antibiotics an effective treatment for corona? |
| 6. | Verbreiten 5G-Mobilfunknetze Corona? | 6. | Do 5G networks spread corona? |
| 7. | Wird Corona durch ein Bakterium verursacht? | 7. | Is corona caused by a bacterium? |
| 8. | Erholt sich die Mehrheit der mit Corona infizierten Menschen wieder davon? | 8. | Do most people who are infected with corona recover? |
